# Supplementary material for: SARS-CoV-2 antibodies and breakthrough infections in the Virus Watch cohort
Source: Nat Commun. 2022 Aug 18;13:4869. doi: 10.1038/s41467-022-32265-5 (PMC9387883; doi:10.1038/s41467-022-32265-5)
Supplement: Supplementary file 1 — Supplementary Information [file 41467_2022_32265_MOESM1_ESM.pdf]

Supplementary Information

Figure S1. Directed acyclic graph for estimating the total effect of anti-S on risk of SARS-CoV-2 Infection

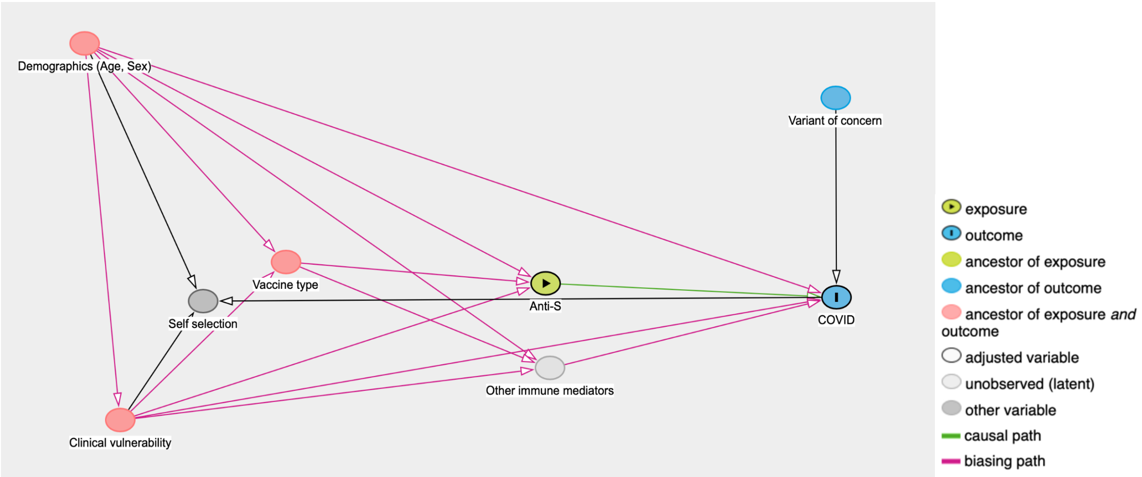

Directed acyclic graph (DAG) displayed created to report our causal assumptions, which illustrates why we conditioned on clinical vulnerability, age, sex and vaccine type to estimate the total effect of anti-S on risk of SARS-CoV-2 infection. Figure created using http://www.dagitty.net/dags.html

Legend text: Vaccine type includes Oxford, AstraZeneca or Pfizer COVID-19 vaccine; Anti-S = SARS-CoV-2 antibodies targeting the spike protein.

Table S1. Demographic and clinical characteristics of individuals in the test negative case-control study.

	Eligible<br>N (%)	Case<br>N (%)	Control<br>N (%)
All	24,189 (100%)	1,832 (100%)	7,328 (100%)
Age group			
18-24	818 (3.4%)	64 (3.5%)	180 (2.5%)
25-44	5,282 (22%)	579 (32%)	1,314 (18%)
45-64	10,552 (44%)	860 (47%)	3,499 (48%)
65+	7,537 (31%)	329 (18%)	2,335 (32%)
Sex			

Female	13,504 (56%)	1,015 (55%)	4,305 (59%)
Male	9,613 (40%)	709 (39%)	2,732 (37%)
Other/Missing	1,072 (4.4%)	108 (5.9%)	291 (4.0%)
Ethnicity			
Black	136 (0.6%)	6 (0.3%)	32 (0.4%)
Mixed	281 (1.2%)	21 (1.1%)	69 (0.9%)
Other Asian	185 (0.8%)	13 (0.7%)	48 (0.7%)
Other/Missing	1,250 (5.2%)	121 (6.6%)	333 (4.5%)
South Asian	649 (2.7%)	72 (3.9%)	132 (1.8%)
White British	19,990 (83%)	1,496 (82%)	6,261 (85%)
White Irish	344 (1.4%)	22 (1.2%)	93 (1.3%)
White Other	1,354 (5.6%)	81 (4.4%)	360 (4.9%)
Clinical vulnerability			
Clinically extremely vulnerable	2,132 (8.8%)	130 (7.1%)	661 (9.0%)
Clinically vulnerable	6,344 (26%)	499 (27%)	1,904 (26%)
Not clinically vulnerable	15,713 (65%)	1,203 (66%)	4,763 (65%)
Time since 2nd dose (days)			
14-76		283 (15%)	1,448 (20%)
77-104		262 (14%)	1,308 (18%)
105-132		363 (20%)	1,348 (18%)
133-160		415 (23%)	1,400 (19%)
161+		509 (28%)	1,824 (25%)
LTLA incidence rate			
0-248		262 (14%)	1,048 (14%)
249-314		351 (19%)	1,404 (19%)
315-384		382 (21%)	1,528 (21%)
385-480		409 (22%)	1,636 (22%)
481+		428 (23%)	1,712 (23%)
2nd dose vaccine type			
BNT162b2	8,835 (37%)	485 (26%)	2,540 (35%)
ChAdOx1	14,764 (61%)	1,315 (72%)	4,604 (63%)
Other/Missing	590 (2.4%)	32 (1.7%)	184 (2.5%)

Table S2. Clinically extremely vulnerable classification

Individuals were categorised as extremely clinically vulnerable using criteria set out by Public Health England and the Department of Health and Social Care as part of the guidance for shielding (https://www.gov.uk/government/publications/guidance-on-shielding-and-protecting-extremely-vulnerable-persons-from-covid-19), which were adapted in line with clinical variables collected through the Virus Watch baseline survey, as follows:

Clinically extremely vulnerable (CEV) criteria as per PHE/DHSC	Inclusion in Virus Watch CEV definition
Solid organ transplant recipients	Included
Cancer undergoing active chemotherapy	Included
Cancers undergoing radical radiotherapy	All radiotherapy included (radical radiotherapy was not ascertained)
Cancer of blood or bone marrow	Included
Immunotherapy or antibody treatments for cancer	Included
Targeted cancer therapies affecting the immune system	Included
Bone marrow or stem cell transplant in last 6 months or still taking immunosuppressive drugs	Included
Severe respiratory conditions including all cystic fibrosis, severe asthma and severe chronic obstructive pulmonary disease (COPD)	Included
Rare diseases that significantly increase the risk of infections (such as severe combined immunodeficiency (SCID), homozygous sickle cell disease)	Included
Immunosuppressive therapies sufficient to significantly increase risk of infection	Included
Problems with spleen, including splenectomy	Included
Down’s syndrome	Not included in CEV as not distinguished from other learning disabilities.

Chronic kidney disease Stage 5 or on renal dialysis	All CKD was included (stage was not ascertained)
Pregnancy with significant heart disease	Included
Others classified as clinically extremely vulnerable	Included

Table S3. Clinically vulnerable classification
Individuals were categorised as clinically vulnerable (CV) using criteria set out by the Joint Committee on Vaccination and Immunisation (https://www.gov.uk/government/publications/priority-groups-for-coronavirus-covid-19-vaccination-advice-from-the-jcvi-30-december-2020), excluding those who met the superseding clinically extremely vulnerable (CEV) criteria. Clinical vulnerability criteria were adapted in line with clinical variables collected through the Virus Watch baseline survey, as follows:

**Clinically vulnerable (CV) criteria as per JCVI**	**Inclusion in Virus Watch CV definition**
chronic respiratory disease, including chronic obstructive pulmonary disease (COPD), cystic fibrosis and severe asthma	Included, except those that met CEV criteria
chronic heart disease (and vascular disease)	Included
chronic kidney disease	Included, except those that met CEV criteria
chronic liver disease	Included
chronic neurological disease including epilepsy	Included
Down’s syndrome	Included as part of broader learning disabilities
Severe and profound learning disability	All learning disabilities included (severity was not ascertained)

Diabetes	Included
Solid organ, bone marrow and stem cell transplant recipients	Not included (included in CEV)
People with specific cancers	Included, except those that met CEV criteria
Immunosuppression due to disease or treatment	Included, except those that met CEV criteria
Asplenia and splenic dysfunction	Not included (included in CEV)
Morbid obesity	Included
Severe mental illness	Included

Declarations of interest

ACH serves on the UK New and Emerging Respiratory Virus Threats Advisory Group. AMJ is Chair of the Committee for Strategic Coordination for Health of the Public Research.
