# Supplementary material for: SARS-CoV-2 antibodies and breakthrough infections in the Virus Watch cohort
Source: Nat Commun. 2022 Aug 18;13:4869. doi: 10.1038/s41467-022-32265-5 (PMC9387883; doi:10.1038/s41467-022-32265-5)
Supplement: Supplementary file 3 — Reporting Summary [file 41467_2022_32265_MOESM3_ESM.pdf]

nature portfolio

Corresponding author(s): Robert Aldridge

Last updated by author(s): Jul 4, 2022

Reporting Summary

Nature Portfolio wishes to improve the reproducibility of the work that we publish. This form provides structure for consistency and transparency in reporting. For further information on Nature Portfolio policies, see our Editorial Policies and the Editorial Policy Checklist.

Statistics

For all statistical analyses, confirm that the following items are present in the figure legend, table legend, main text, or Methods section.

n/a	Confirmed	
☐	☒	The exact sample size (*n*) for each experimental group/condition, given as a discrete number and unit of measurement
☐	☒	A statement on whether measurements were taken from distinct samples or whether the same sample was measured repeatedly
☐	☒	The statistical test(s) used AND whether they are one- or two-sided<br>*Only common tests should be described solely by name; describe more complex techniques in the Methods section.*
☐	☒	A description of all covariates tested
☐	☒	A description of any assumptions or corrections, such as tests of normality and adjustment for multiple comparisons
☐	☒	A full description of the statistical parameters including central tendency (e.g. means) or other basic estimates (e.g. regression coefficient) AND variation (e.g. standard deviation) or associated estimates of uncertainty (e.g. confidence intervals)
☐	☒	For null hypothesis testing, the test statistic (e.g. *F*, *t*, *r*) with confidence intervals, effect sizes, degrees of freedom and *P* value noted<br>*Give P values as exact values whenever suitable.*
☒	☐	For Bayesian analysis, information on the choice of priors and Markov chain Monte Carlo settings
☒	☐	For hierarchical and complex designs, identification of the appropriate level for tests and full reporting of outcomes
☐	☒	Estimates of effect sizes (e.g. Cohen's *d*, Pearson's *r*), indicating how they were calculated

Our web collection on statistics for biologists contains articles on many of the points above.

Software and code

Policy information about availability of computer code

Data collection	Survey data were collected from participants using REDCap 12.4.0 https://www.project-redcap.org/
Data analysis	Data analyses were conducted in R R 4.0.3 and Python 3.6.0

For manuscripts utilizing custom algorithms or software that are central to the research but not yet described in published literature, software must be made available to editors and reviewers. We strongly encourage code deposition in a community repository (e.g. GitHub). See the Nature Portfolio guidelines for submitting code & software for further information.

Data

Policy information about availability of data

All manuscripts must include a data availability statement. This statement should provide the following information, where applicable:

- Accession codes, unique identifiers, or web links for publicly available datasets
- A description of any restrictions on data availability
- For clinical datasets or third party data, please ensure that the statement adheres to our policy

Data used in this study are available from the Office of National Statistics Secure Research Service.

Field-specific reporting

Life sciences study design

All studies must disclose on these points even when the disclosure is negative.

Sample size	A-priori sample size calculations were carried out for the main Virus Watch cohort as reported in our study protocol http://dx.doi.org/10.1136/bmjopen-2020-048042.
Data exclusions	Antibody results were excluded after individuals reported a third booster COVID-19 vaccination and in individuals who were anti-N positive prior to 1st July 2021.
Replication	To replicate our test negative case control finding, we undertook a separate analysis comparing risk of breakthrough of ChAdOx1 compared to BNT162b2 using a target trial comparative effectiveness observational study design. The analysis was performed by a separate analyst and confirmed the reproducibility of our findings.
Randomization	Participants were not allocated into experimental groups.
Blinding	Participants and researchers were not blinded as this was a cohort study conducted in the community and not a randomised controlled trial of a new intervention that would require blinding.

Reporting for specific materials, systems and methods

We require information from authors about some types of materials, experimental systems and methods used in many studies. Here, indicate whether each material, system or method listed is relevant to your study. If you are not sure if a list item applies to your research, read the appropriate section before selecting a response.

Materials & experimental systems

n/a	Involved in the study	
☐	☒	Antibodies
☒	☐	Eukaryotic cell lines
☒	☐	Palaeontology and archaeology
☒	☐	Animals and other organisms
☐	☒	Human research participants
☐	☒	Clinical data
☒	☐	Dual use research of concern

Methods

n/a	Involved in the study	
☒	☐	ChIP-seq
☒	☐	Flow cytometry
☒	☐	MRI-based neuroimaging

Antibodies

Antibodies used	We measured antibody titres targeting the spike (S) protein (anti-S) in the context of seronegativity for SARS-CoV-2 anti-Nucleocapsid (anti-N) which is associated with natural infection. Sera were tested using Elecsys anti-S and anti-N electro-chemiluminescent immunoassays (Roche Diagnostics, Basel, Switzerland).
Validation	Performance characteristics of five immunoassays for SARS-CoV-2: a head-to-head benchmark comparison https://pubmed.ncbi.nlm.nih.gov/32979318/

Human research participants

Policy information about studies involving human research participants

Population characteristics	The Virus Watch study is a household community cohort of acute respiratory infections in England & Wales that started recruitment in June 2020, with participants providing informed consent. In this analysis, individuals were included in this analysis if they underwent antibody testing (anti-N and anti-S) and had a valid result between 1st July 2021 and 24th October 2021. We included people over the age of 18 who were seronegative for SARS-CoV-2 anti-Nucleocapsid (anti-N). In total, 58,679 participants of age (0 - 98 years, mean age 48) took part in the study, with more women (47%) than men (37%) and 69% of people with White British ethnicity. In order to boost the minority ethnici population within the cohort, we sent targeted letters via GP clinics that included £10 voucher incentives to sign up.
Recruitment	We used a range of methods including the Royal Mail Post Office Address File to generate a random list of residential address lists that were sent recruitment postcards, we placed social media adverts on Facebook and Twitter and sent SMS messages and letters to participants from their General Practitioners.
Ethics oversight	The Virus Watch study has been approved by the Hampstead NHS Health Research Authority Ethics Committee. Ethics approval number - 20/HRA/2320.

Note that full information on the approval of the study protocol must also be provided in the manuscript.

Clinical data

Policy information about clinical studies

All manuscripts should comply with the ICMJEguidelines for publication of clinical research and a completedCONSORT checklist must be included with all submissions.

Clinical trial registration	The study is registered with ISRCTN here: https://doi.org/10.1186/ISRCTN32077121
Study protocol	We have published a study protocol here: http://dx.doi.org/10.1136/bmjopen-2020-048042
Data collection	The Virus Watch study is a household community cohort of acute respiratory infections in England & Wales that started recruitment in June 2020 and is ongoing. We included self-reported vaccination status, and vaccine type, collected weekly from 11 January 2021 onwards. Age, sex, ethnicity and geographical region were derived from participants' responses to demographic questions at study baseline.
Outcomes	We considered two primary outcomes. First, SARS-CoV-2 anti-S titre in the context of seronegativity for SARS-CoV-2 anti-N. Second, SARS-CoV-2 positive tests confirmed using PCR or rapid lateral flow antigen tests. We defined breakthrough SARS-CoV-2 infection as a positive test (PCR or LFD) after being fully vaccinated at least 14 days following the second dose of BNT162b2 or ChAdOx1. We only included individuals with anti-S levels measured at least 14 days prior to breakthrough infection to ensure anti-S levels used upon cohort entry were not inflated by early asymptomatic breakthrough infections that develop into symptomatic infections. For this current analysis, we did not examine the presence or absence of symptoms in the context of a positive SARS-CoV-2 test.
